# Evaluation of intestinal microbiota, short-chain fatty acids, and immunoglobulin a in diversion colitis

**DOI:** 10.1016/j.bbrep.2020.100892

**Published:** 2020-12-30

**Authors:** Kentaro Tominaga, Atsunori Tsuchiya, Takeshi Mizusawa, Asami Matsumoto, Ayaka Minemura, Kentaro Oka, Motomichi Takahashi, Tomoaki Yosida, Yuzo Kawata, Kazuya Takahashi, Hiroki Sato, Satoshi Ikarashi, Kazunao Hayashi, Ken-ichi Mizuno, Yosuke Tajima, Masato Nakano, Yoshifumi Shimada, Hitoshi Kameyama, Junji Yokoyama, Toshifumi Wakai, Shuji Terai

**Affiliations:** a Division of Gastroenterology and Hepatology, Graduate School of Medical and Dental Sciences, Niigata University, Niigata, Japan; b Research Department, R&D Division, Miyarisan Pharmaceutical Co., Ltd., Saitama, Japan; c Division of Digestive and General Surgery, Graduate School of Medical and Dental Sciences, Niigata University, Niigata, Japan

**Keywords:** Diversion colitis, Microbiota, Short-chain fatty acids, Immunoglobulin A, DC, diversion colitis, OA, organic acid, SCFAs, short-chain fatty acids, AA, acetic acid, BA, butyric acid, PA, propionic acid, IgA, immunoglobulin A

## Abstract

It is reported that an increase in aerobic bacteria, a lack of short-chain fatty acids (SCFAs), and immune disorders in the diverted colon are major causes of diversion colitis. However, the precise pathogenesis of this condition remains unclear. The aim of the present study was to examine the microbiota, intestinal SCFAs, and immunoglobulin A (IgA) in the diverted colon. Eight patients underwent operative procedures for colostomies. We assessed the diverted colon using endoscopy and obtained intestinal samples from the diverted colon and oral colon in these patients. We analyzed the microbiota and SCFAs of the intestinal samples. The bacterial communities were investigated using a 16S rRNA gene sequencing method. The microbiota demonstrated a change in the proportion of some species, especially *Lactobacillus*, which significantly decreased in the diverted colon at the genus level. We also showed that intestinal SCFA values were significantly decreased in the diverted colon. Furthermore, intestinal IgA levels were significantly increased in the diverted colon. This study was the first to show that intestinal SCFAs were significantly decreased and intestinal IgA was significantly increased in the diverted colon. Our data suggest that SCFAs affect the microbiota and may play an immunological role in diversion colitis.

## Highlights

• Anaerobic bacteria like *Lactobacillus* significantly decreased in the diverted colon.
• Fecal short-chain fatty acids significantly decreased in the diverted colon.
• Acetic acid also decreased significantly in the diverted colon.
• Fecal immunoglobulin A levels were significantly increased in the diverted colon.

## 1 Introduction

Diversion colitis (DC) was first described by Morson et al., in 1974 as nonspecific inflammation in the diverted colon [1]. Glotzer et al. termed this inflammation “diversion colitis” in 1981 [2]. A prospective study reported that almost all cases show colitis, as evidenced by endoscopic analyses, 3–36 months after a colostomy [3]. Most patients are asymptomatic; however, approximately one-third of patients may exhibit various symptoms of DC, such as abdominal discomfort, tenesmus, anorectal pain, mucous discharge, and rectal bleeding [4,5].

It has been reported that an increase in aerobic bacteria, a lack of short-chain fatty acids (SCFAs), and immune disorders of the diverted colon are major causes of DC [6]. However, the precise pathogenesis of this condition remains unclear. While there are a few reports on the microbiota in DC in humans, the relationship between the microbiota and SCFAs or immunoglobulin A (IgA) remains elusive.

The aim of the present study was to evaluate the intestinal microbiota, SCFAs, and IgA in the diverted colon and to elucidate the pathogenesis of DC.

## 2 Material and methods

### 2.1 Subjects and study protocol

Written informed consent was obtained from the patients for publication of the report and accompanying images. The study was reviewed and approved by the Institutional Review Board of Niigata University.

The subjects were 8 patients (65.9 ± 10.4 years) (male: female = 1:7), who had undergone stoma surgery within the previous 1–40 months. Five of these patients had a history of colon cancer, one had rectovaginal septum cancer, one had ovarian cancer, and one had a retroperitoneal abscess. These patients all underwent a colostomy (Table 1). None of the patients was administered with antibiotics or probiotics for one month leading up to the date of the colonoscopy.

**Table 1:** Patient characteristics.

Case (No)	Age (yrs)	Sex	Diagnosis	Operative procedure	Post-surgical treatment for primary disease (anti-cancer drugs or antibiotics)	Symptom	UCEIS	Postoperative period
1	62	M	Rectal Cancer	Low anterior resection + Colostomy	none	none	1	10 M
2	49	F	Rectal Cancer	Low anterior resection + Colostomy	XELOX chemotherapy (6 M)	none	2	6 M
3	66	F	Rectal Cancer	Low anterior resection + Colostomy	FOLFOX chemotherapy (6 M)	none	2	6 M
4	86	F	Sigmoid colon Cancer	Low anterior resection + Colostomy	none	none	1	1 M
5	67	F	Rectovaginal septum cancer	Transverse colostomy	Ceftriaxone (2 W)	mucous and<!--Soft-enter Run-on-- > bloody stool	3	40 M
6	74	F	Ovarian Cancer	Transverse colostomy	Sulbactam/Cefoperazone (1 W) + Piperacillin/Tazobactam (1 W)	mucous stool	3	18 M
7	56	F	Rectal Cancer	Low anterior resection + Colostomy	none	none	1	1 M
8	67	F	Retroperitoneal abscess	Left hemicolectomy + Transverse colostomy	Piperacillin/Tazobactam (1 M)	mucous stool	2	16 M

We assessed the diverted colon endoscopically, and evaluated the severity using the ulcerative colitis endoscopic index of severity (UCEIS). We assessed the intestinal microbiota of the diverted colon and oral colon using a next-generation sequencer (Illumina MiSeq). We conducted 16S rRNA gene sequencing. The composition of intestinal microbiota was evaluated using quantitative insights into microbial ecology (QIIME), and β-diversity was measured using UniFrac-distances analysis.

We also measured the composition of intestinal organic acids, SCFA, and IgA through enzyme-linked immunosorbent assay (ELISA) in six patients.

### 2.2 Intestinal sample collection

Intestinal samples (approximately 100 mg) were suspended in 900 μL of guanidine thiocyanate solution (100 mM Tris–HCl [pH 9.0], 40 mM EDTA, and 4 M guanidine thiocyanate) and frozen at −80 °C until further analysis [7].

### 2.3 DNA preparations from intestinal samples

The collected samples were sent to the laboratory of Miyarisan Pharmaceutical Co., Ltd. and stored at −20 °C. DNA was extracted from collected intestinal samples using a glass bead extraction method and purified, according to a previously reported method [8]. The amount of DNA was determined using a Quanti Fluor dsDNA System and Quantus Fluorometer (Promega, Madison, WI, USA).

### 2.4 PCR amplification and analysis of 16S rRNA sequences

The V3–V4 region of the 16S rRNA gene was PCR amplified from stool DNA samples using a TaKaRa Ex Taq Hot Start PCR mixture (Takara Bio, Shiga, Japan). The primers used for PCR amplification were 341F and 785R, which contained the Illumina index and sequencing adapter overhangs [9]. PCR assays were performed using a TaKaRa PCR Thermal Cycler Dice Touch device (Takara Bio, Shiga, Japan) with the following parameters: initial denaturation at 98 °C for 30 s, followed by 35 cycles of 98 °C for 10 s and 60 °C for 30 s, with a final extension step at 72 °C for 5 min. The PCR products were purified and size selected using SPRIselect (Beckman Coulter, Brea, CA, USA). DNA concentrations were quantified with a QuantiFluor dsDNA System and Quantus Fluorometer (Promega, Madison, WI, USA), and equal amounts of purified PCR products were pooled for subsequent Illumina MiSeq sequencing. Sequencing was carried out with a Miseq Regent Kit V3 (600 cycles) (Illumina, San Diego, CA, USA), according to the manufacturer's instructions. Sequence processing and quality assessment were performed using the QIIME package (version 1.8.0) (http://qiime.org), an open source software created to address the problem of obtaining sequencing data from raw sequences for interpretation and database deposition [10]. To obtain an overall diversity analysis for subsequent comparative and statistical evaluations, we merged the Biological Observation Matrix (BIOM) tables provided by QIIME into a unique biom table using a script included in the QIIME package. Paired-end reads were merged using the Fastq-join script in ea-utils with the parameters m = 6 and P = 20, then quality filtered using QIIME's script split_libraries_fastq.py (r = 3, P = 0.75, q = 20, n = 0). De novo and reference-based chimera detection and removal were performed using USEARCH v6.1 with the Greengenes v13.8 database. Operational taxonomic units (OTUs) were chosen using an open reference OTU-picking pipeline against the 97% identity of the pre-clustered Greengenes v13.8 database using UCLUST. According to the manufacturer, a QIIME alpha diversity analysis script is used to perform rarefaction analysis by subsampling the OTUs biom table based on the minimum rarefaction depth value chosen by the user depending on the minimum number of sequences/samples obtained. For our subset, this value was 3957. Then, using different metrics, alpha diversity was computed for each rarefied OTU table. We used three non-phylogeny-based metrics: observed species, chao 1, and the Shannon index. After performing the rarefaction evaluation, the QIIME beta diversity analysis script was used to compute beta diversity with the rarefied OTUs table using different metrics. We used a non-phylogeny-based metric (Bray–Curtis metric) as well as phylogeny-based metrics (unweighted and weighted UniFrac) [11]. Finally, the script was used to obtain a distance metric to compute the principal coordinate analysis (PCoA) and convert it into plots for results visualization.

### 2.5 Analysis of intestinal organic acids

For determination of organic acids, 0.1 g of feces was placed in a 2.0 mL tube with zirconia beads and suspended in MilliQ. Samples were heated at 85 °C for 15 min, vortexed at 5 m/s for 45 s using FastPrep 24 5G (MP Biomedicals, CA, USA), and centrifuged at 15,350×*g* for 10 min. The supernatant was filtered through a 0.2 μm filter. Organic acids (acetic acid, propionic acid, butyric acid, iso-butyric acid, succinic acid, lactic acid, formic acid, valeric acid, and iso-valeric acid) in feces were measured using high-performance liquid chromatography (Prominence, SHIMADZU, Kyoto, Japan) using a post column reaction with a detector (CDD-10A, SHIMADZU, Kyoto, Japan), tandemly arranged two columns (Shim-pack SCR-102(H), 300 mm × 8 mm ID, SHIMADZU, Kyoto, Japan), and a guard column (Shim-pack SCR-102(H), 50 mm × 6 mm ID, SHIMADZU, Kyoto, Japan). The system was used with a mobile phase (5 mM *p*-toluenesulfonic acid) and a reaction solution (5 mM *p*-toluenesulfonic acid, 100 μM EDTA, and 20 mM Bis-Tris). The flow rate and oven temperature were 0.8 mL/min and 45 °C, respectively. The detector cell temperature was maintained at 48 °C.

### 2.6 Analysis of intestinal IgA

The quantification of intestinal IgA was performed using Cosmo Bio Co., Ltd. (Tokyo, Japan). Intestinal IgA content was quantified through ELISA using a mouse IgA ELISA Quantitation Set (Bethyl Laboratories, Inc., Montgomery, TX, USA). Approximately 50 mg of feces was used for IgA analysis, and the values were expressed as values per feces weight.

### 2.7 Statistical analysis

All quantitative data are expressed as mean ± SE. A two-sided Student's two-sample *t*-test was used for statistical analyses with the SPSS statistical package, version 24.0 (IBM SPSS Japan Inc., Tokyo, Japan). In this study, while values of P < 0.05 were considered statistically significant, if significance did not remain after correction using an optimized false discovery rate approach to account for false-positive results, presented as q values, they are instead described as tendencies. The significance of each PCoA plot was analyzed using permutational multivariate analysis of variance, a non-parametric test similar to analysis of variance that does not require the data to be normally distributed, and uses distance metrics to confirm the strength and statistical significance of sample groupings [12]. We used 999 Monte Carlo permutations in QIIME to assess statistical significance between group diversity metrics.

## 3 Results

### 3.1 Endoscopic evaluation and symptoms

Eight patients underwent stoma surgery within 1–40 months prior to this study. We performed an endoscopy for all patients and evaluated the inflammation of the diverted colon using the UCEIS. While the method for assessing inflammation of DC has not yet been established, it is often evaluated using the UCEIS score.

In all patients, mild DC (UCEIS = 1 to 3) was detected by colonoscopy (Table 1). Details of inflammation were UCEIS = 1 in 3 patients, UCEIS = 2 in 3 patients, and UCEIS = 3 in 2 patients. Only three patients presented with symptoms of mucous, bloody stool, and tenesmus, and all three had surgery over a year prior. Patients who had a longer period after surgery tended to have more colonic inflammation and were more symptomatic (Table 1).

### 3.2 Intestinal microbiota

**Fig. 1:**
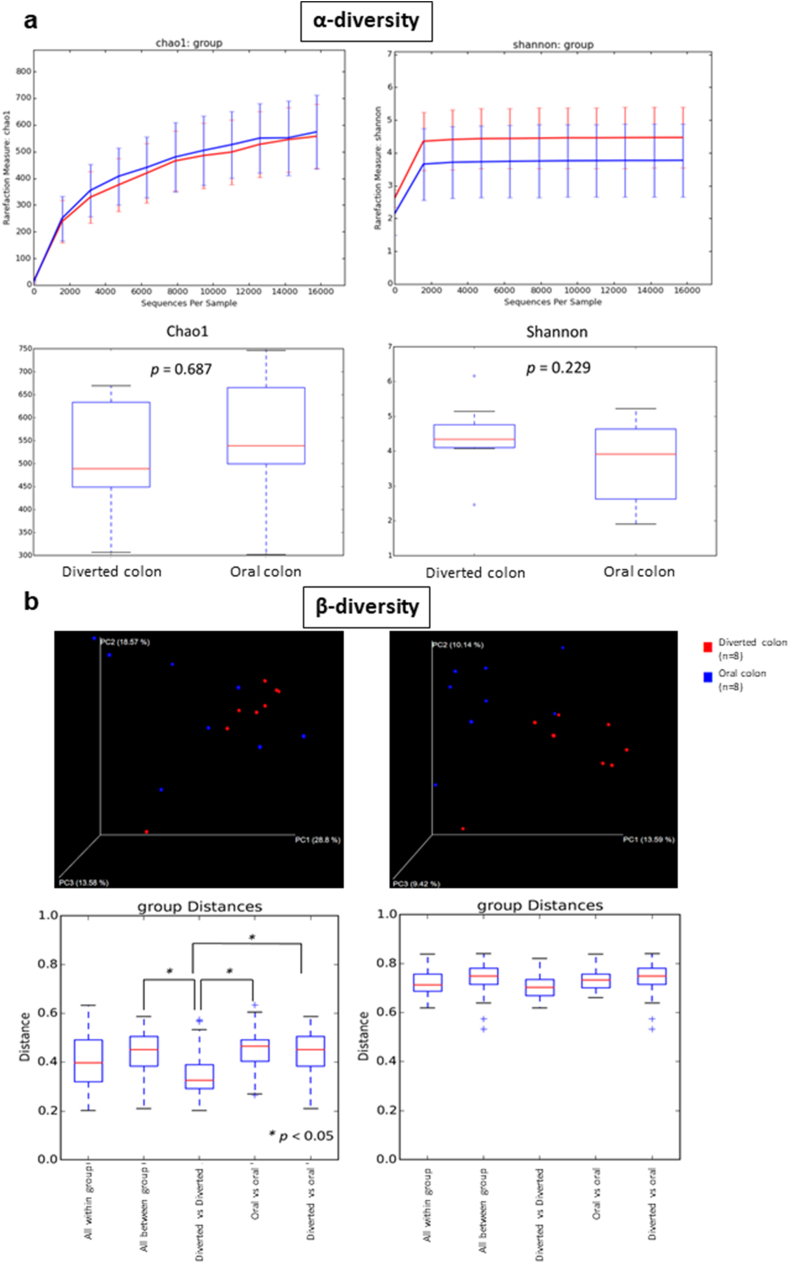
**(a)** Theα-diversity data of this study. Bars show the SD of the data. Data were analyzed using the Mann–Whitney *U* test (vs. Pre). **(b)** The β -diversity data of this study. Bars show the SD of the data. Data were analyzed using the Mann–Whitney *U* test (vs. Pre) followed by Benjamini–Hochberg (*: P < 0.05).

The α-diversity data showed that there was no significant difference (Fig. 1a) among the microbiota in the diverted colon and in the oral colon. Regarding the β-diversity, there was a significant difference between “diverted colon vs. diverted colon” and “oral colon vs. oral colon” and between “diverted colon vs. diverted colon” and “diverted colon vs. oral colon” (Fig. 1b). These results indicate that the difference in the composition ratio of the microbiota in the oral colon is larger than that in the diverted colon, and the difference in the composition ratio of the microbiota between these two groups was larger than that in the diverted colon group. The β-diversity data showed that the microbiota in the diverted colon formed a cluster, which was significantly different from the microbiota in the oral colon.

**Fig. 2:**
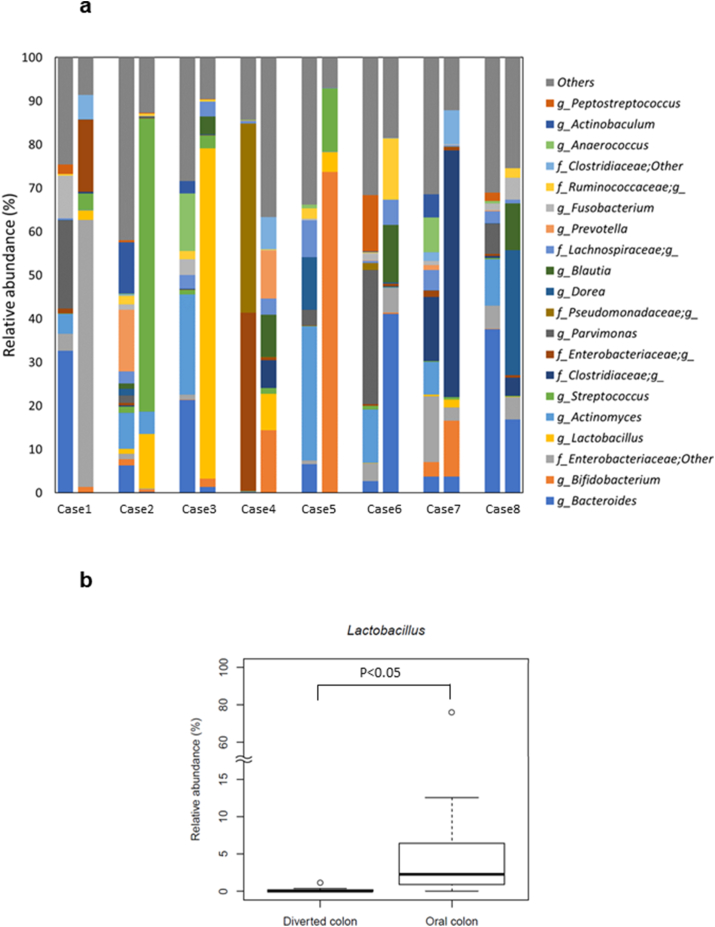
**(a)** The analysis of the intestinal microbiota. The comparison of microbiota between the diverted colon and the oral colon of the patients, who underwent colostomy. **(b)** Significant differences were detected in the numbers of *Lactobacillus* (at genus level) (P < 0.05). Bars show the SD of the data. Data were analyzed using the Mann–Whitney *U* test (vs. Pre) followed by the Benjamini–Hochberg procedure (*P < 0.05, **p < 0.01).

Regarding the intestinal microbiota at the genus level, the serial intestinal microbiota showed an increase in *Actinomyces* (P < 0.05), *Anaerococcus* (P < 0.05), *Corynebacterium* (P < 0.01), *Peptoniphilus* (P < 0.05), and *Porphyromonas* (P < 0.01), with significantly different intestinal organisms in the diverted colon compared to those in the oral colon (Fig. 2a). Moreover, we detected a decrease in *Lactobacillus* (P < 0.05) and *Granulicatella* (P < 0.05), which were significantly changed in the diverted colon compared to those in the oral colon (Fig. 2b).

### 3.3 Intestinal **SCFA and IgA**

**Fig. 3:**
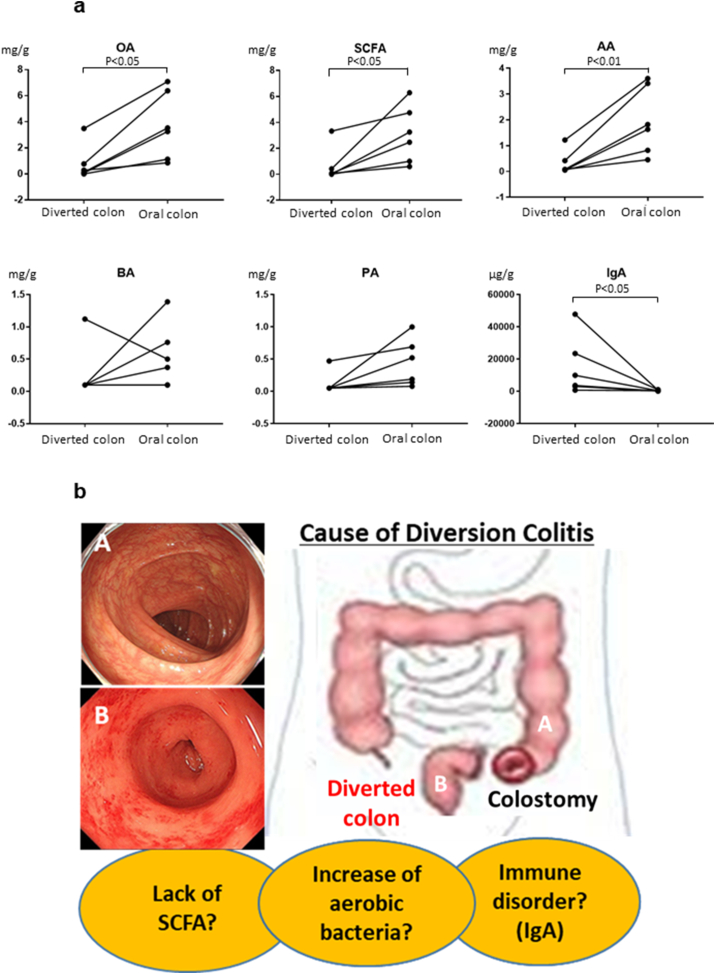
**(a)** The comparison of intestinal elements between the diverted colon and oral colon. Significant differences were detected for OA (P < 0.05), SCFAs (P < 0.05), AA (P < 0.01), and IgA (P < 0.05). OA; organic acid, SCFA; short-chain fatty acids, AA; acetic acid, BA; butyric acid, PA; propionic acid, IgA; immunoglobulin A. **(b)** Schematic of diversion colitis.

The comparison of intestinal organic acid (OA), SCFAs, and acetic acid (AA) in the diverted colon and the oral colon showed that there were significant differences in six patients, with averages (diverted colon vs oral colon, mean ± SD) of 0.79 ± 1.2 vs. 3.71 ± 2.4 (P < 0.05) for OA, 0.65 ± 1.2 vs. 3.06 ± 2.0 (P < 0.05) for SCFAs, and 0.45 ± 0.3 vs. 1.96 ± 1.2 (P < 0.01) for AA (Fig. 3a).

However, there were no significant differences in intestinal butyric acid (BA) and propionic acid (PA) values between these groups.

The comparison of intestinal IgA in the diverted colon and oral colon showed that there was a significant difference in six patients, with an average (diverted colon vs oral colon, mean ± SD) of 14821 ± 8221 vs. 520 ± 339 (P < 0.05) (Fig. 3a). Interestingly, the diverted colon had a higher IgA than the oral colon in all cases.

## 4 Discussion

The precise pathogenesis of DC remains unclear. It is considered that an increase in aerobic bacteria, a lack of SCFAs, and immune disorders in the diverted colon are major causes of DC. We have summarized the possible disease pathogenesis in Fig. 3b.

There are several reports on the microbiota in DC; Neut et al. showed a decrease in anaerobes of the diverted colon [13]. Se-Jin Baek et al. reported a decrease in anaerobes, notably in *Lactobacillus* and *Bifidobacterium* [14]. These reports are comparisons between patients with DC and normal subjects.

Almost all of our patients had mild inflammation; and therefore, we could not derive a correlation between the intensity of inflammation and the microbiota. Considering the diversity in the composition of the microbiota in different individuals, in our study, we instead compared the microbiota in the diverted colon with that in the oral colon of the same individual. We concluded that *Lactobacillus* was predominantly low in the diverted colon. *Lactobacillus* has been reported to promote regulatory T cell differentiation and suppress enteritis [15]; therefore, it is considered one of the causes of DC.

SCFAs are produced mainly through saccharolytic fermentation of carbohydrates that escape digestion and absorption in the small intestine [16]. The pathways of SCFA production are relatively well understood and have been recently described in detail [17]. However, to date, there have been no reports on SCFA measurements in DC. In this study, we showed that SCFAs were significantly reduced in the diverted colon. The complex metabolic cross-feeding relationships among the microbial populations is influenced by the environment, such as changes in the nutrient supply, particularly in complex environments, such as the human lower gut [18]. SCFAs are the major fuel source for the intestinal epithelium. Bacteria produce SCFAs as byproducts of carbohydrate fermentation in the colonic lumen, and SCFAs provide the primary energy source for colonic mucosal cells [19]. Their absence in the diverted colon may cause mucosal atrophy and inflammation.

IgA is the main antibody isotype secreted into the intestinal lumen, and it plays a critical role in defense against pathogens and in the maintenance of intestinal homeostasis [20]. However, how secreted IgA regulates the gut microbiota is not completely understood. It has been reported that intestinal IgA selectively binds harmful bacteria to eliminate them from the gut microbiota [21]. On the other hand, several reports have demonstrated that IgA binding could facilitate gut colonization by bound bacteria, rather than suppressing their growth [22,23]. There are no previous reports on intestinal IgA in DC. Here, we showed that intestinal IgA was increased in the diverted colon.

The fundamental treatment of DC is intestinal anastomosis. Therapeutic options other than surgical procedures include 5-aminosalicylic acids, glucocorticoids, antibiotics, SCFAs, and intestinal microbiota transplantation (FMT) [6,24]. The treatment of DC with SCFAs has shown some positive results, with Harig demonstrating improved symptoms and endoscopic inflammatory changes [24]. Komorowski et al. reported similar results in four patients with DC following SCFA irrigation [25]. In recent years, several studies on the usefulness of SCFAs, including butyrate, have been reported [26,27]. Cristina et al. [28] proposed that butyrate enemas might prevent the atrophy of the diverted colon/rectum, thus improving the recovery of tissue integrity. We also reported that the usefulness of FMT for DC and intestinal microbiota was important for homeostasis of the colon [29].

Our results suggest that SCFAs (including AA) affects the intestinal microbiota, which may play a role in the immunity of the diverted colon. This study was the first to show that intestinal SCFAs were significantly decreased and intestinal IgA was significantly increased in the diverted colon. Our results were also consistent with those of previous reports in which the proportion of *Lactobacillus* was significantly decreased in diverted colon. Our data suggest that SCFAs affect the microbiota and may play a role in improving DC. However, further studies, such as a comparison of the microbiota between patients with and without diversion colitis, on a large scale, are essential to elucidate the pathogenesis of DC.

## Ethics approval and consent to participate

The study was reviewed and approved by the Institutional Review Board of Niigata University.

## Consent for publication

Written informed consent was obtained from the patients for publication of the report and accompanying images.

## Availability of data and materials

Not applicable.

## Conflicts of interest

These authors disclose the following: Shuji Terai received research funding from 10.13039/100016567Miyarisan Pharmaceutical Co., Ltd. The remaining authors have no conflicts of interest to disclose.

## Funding

This work was supported by Takeda Japan Medical Office Funded Research Grant 2018, Tsukada Medical Funded Research Grant, Grant-in-Aid for Young Scientific Research (19K17393) from the Ministry of Education, Science, Technology, Sports, and Miyarisan Pharmaceutical Co., Ltd.

## Author's contributions

KT, AT, TM, TY, YK, KT, HS, SI, KH, KM, YT, MN, YS, HK, and JY diagnosed and drafted the manuscript. AM, AM, KO, MT, TW, and ST analyzed data. All authors critically reviewed the manuscript and approved the final draft.

## Declaration of competing interest

The authors declare the following financial interests/personal relationships which may be considered as potential competing interests:

The authors disclose the following: Shuji Terai received research funding from 10.13039/100016567Miyarisan Pharmaceutical Co., Ltd. Asami Matsumoto, Ayaka Minemura, Kentaro Oka and Motomichi Takahashi are employees of Miyarisan Pharmaceutical Co., Ltd.The remaining authors disclose no conflicts.

Please note that all Biochemical and Biophysical Research Communications authors are required to report the following potential conflicts of interest with each submission. If applicable to your manuscript, please provide the necessary declaration in the box above.

(1) All third-party financial support for the work in the submitted manuscript.
(2) All financial relationships with any entities that could be viewed as relevant to the general area of the submitted manuscript.
(3) All sources of revenue with relevance to the submitted work who made payments to you, or to your institution on your behalf, in the 36 months prior to submission.
(4) Any other interactions with the sponsor of outside of the submitted work should also be reported. (5) Any relevant patents or copyrights (planned, pending, or issued).
(6) Any other relationships or affiliations that may be perceived by readers to have influenced, or give the appearance of potentially influencing, what you wrote in the submitted work. As a general guideline, it is usually better to disclose a relationship than not.
